# Editorial: Diversity, inclusion, and prejudice in the military

**DOI:** 10.3389/fpsyg.2024.1426561

**Published:** 2024-05-23

**Authors:** Adelheid A. M. Nicol, Cindy Suurd Ralph, Kalee De France, Harris R. Lieberman

**Affiliations:** ^1^Royal Military College of Canada, Military Psychology and Leadership Department, Kingston, ON, Canada; ^2^The University of British Columbia, Okanagan Campus, Kelowna, BC, Canada; ^3^US Army Research Institute of Environmental Medicine (USARIEM), Natick, MA, United States

**Keywords:** gender, veterans, combat, armed forces, contact theory, leadership, tokenism, integration

## Introduction

Many military organizations are navigating a challenging and dynamic period as they strive to include underrepresented populations such as women and individuals with diverse gender and racial identities within their ranks. This Research Topic on *Diversity, inclusion, and prejudice in the military* was designed to illuminate the issues and experiences associated with this form of military integration.

This Research Topic contains seven articles, four of which address issues surrounding gender. This focus may reflect researchers' and nations' commitment to the United Nations Security Council (2000, 2015) which recognized the importance of integrating more women in the military, in prevention of conflict, resolution of conflict, and maintaining and building peace and security as women are more adversely affected by armed conflict. Furthermore, it highlights the difficulties that militaries have experienced in increasing representation of women in the military (Williams et al., 2024). Two papers within this Research Topic examined approaches to integrating women in the military. Tait-Signal and Febbraro, using qualitative studies, highlighted the resistance faced by military gender experts and the challenges they encounter to be viewed as legitimate contributors to military efforts. Surveying a male military sample, as well as male and female civilian samples, Nicol and Mayrand Nicol found that having more experiences with women in leadership positions was related to more favorable attitudes toward women in the military. They also report that the role of intermediary variables, such as intergroup anxiety, empathy, and perspective-taking, differed depending upon the sample studied, suggesting that the mechanisms to explain how contact influences attitudes may differ depending upon a variety of contextual variables. Together, these two papers provide preliminary evidence for measures military organizations can take to enhance the integration of women in the military: having more women in leadership positions and improving the training and education provided to gender advisors in the military appears to improve outcomes at the individual and system-wide level.

Additionally, two papers within this Research Topic examined women's experiences within the military and highlighted difficulties of integration of women in a male-dominated culture (Pendlebury, 2020). The article “Caught in the Crossfire” –Women Veterans' Testimonies regarding excessively violent acts committed in combat zones' by HaCohen and Amir focused on gendered inequality and oppression against women in the Israeli Defense Forces. Qualitative methodologies were used to analyze retrospective testimonies of 50 IDF women veterans, and identified themes of internalized gender oppression, identification with the aggressor, and moral objection rooted in a feminine perspective. Hendel et al. studied active female Canadian Armed Forces members' views regarding their experiences serving in the combat arms. Many expressed favorable attitudes and perceived benefits to working in the military. However, the environment also posed demands such as working in a heteronormative culture, dealing with tokenism, and strains on mental and physical health. These papers provide insight into both positive and negative elements surrounding experiences of women within the military, as well as novel glimpses into the inner tension experienced by many women in military professions worldwide as they navigate between two extremes: victims of male dominance, or aggressors within powerful military systems.

Diversity integration in the military goes beyond gender integration and considers the experiences of minority group members in the military as they experience challenges of poorer health and greater experiences with discrimination (Salem et al., 2023). Two papers in this Research Topic examined minority members' experiences. Examining survey data, Peach et al. found that being a member of a minority group in the Canadian military was related to low feelings of inclusion, which has implications for job retention, satisfaction, and commitment. The paper by Sabar et al. documents the consequences of military service on the integration of children of undocumented migrant workers from countries such as the Philippines into Israeli society in the 1990s. These children were denied citizenship but some were granted permanent residency status and were therefore required to complete compulsory military service. If an individual completed 1 year of service in the military, they were granted citizenship and their parents became legalized residents. This paper documents, based on mixed methods, the extent military service contributed to individuals' integration into Israeli society. Overall, it appears that military service was evaluated positively, and participants believed it helped them overcome the discrimination and marginalization migrants often experience.

Organizational culture change efforts within militaries seek to create more inclusive environments that go beyond heteronormative masculine ideals, by reducing sexual misconduct and inequalities (Deng et al., 2023). Resistance in integrating women into the military highlights the importance of addressing factors that can block organizational culture change efforts (van Douwen et al., 2022). de Grandpré et al.'s work speaks to the broader issues of increasing diversity and changing heteronormative practices within the military by examining attitudes toward organizational culture change. The study found that military members' levels of empathy and feelings of belongingness can impact their support for organizational culture change efforts and that these variables can be influenced by the organization through training programs and enacted leadership.

## Conclusion

The past decades have seen many cultural changes internationally and domestically, but more changes are necessary as the percentage of women and minorities in the military are not representative of their populations (NATO, 2020; Salem et al., 2023). Understanding the impact of their low numbers on civilian and military members, factors that influence attitude change, and understanding hurdles that can slow change are key to addressing the challenges that militaries face in their efforts to improve diversity and inclusion and reduce prejudice.

## Author contributions

AN: Conceptualization, Writing – original draft, Writing – review & editing. CS: Writing – original draft, Writing – review & editing. KD: Writing – original draft, Writing – review & editing. HL: Writing – original draft, Writing – review & editing.

## References

[B1] Deng M. E. Nicol A. A. M. Ralph C. S. (2023). Masculine conformity and social dominance's relation with organizational culture change. Armed Forces Soc. 22:8522. 10.1177/0095327X231178522

[B2] NATO (2020). Summary of National Reports 2020 of NATO Member and Partner Nations to the NATO Committee on Gender Perspective. Available online at: https://www.nato.int/nato_static_fl2014/assets/pdf/2023/6/pdf/2020-summary-national-reports.pdf (accessed April 15, 2024).

[B3] Pendlebury J. (2020). “This is a man's job”: challenging the masculine “warrior culture” at the U.S. Air force academy. Armed Forces Soc. 46, 163–184. 10.1177/0095327X18806524

[B4] Salem K. Randles R. Sapre B. Finnegan A. (2023). Experiences of ethnic minority personnel in the armed forces: a systematic review. J. Militar. Vet. Family Health 9, 5–14. 10.3138/jmvfh-2022-001938144408

[B5] United Nations Security Council (2000). Resolution 1325. Available online at: https://digitallibrary.un.org/record/426075?ln=enandv=pdf (accessed April 15, 2024).

[B6] United Nations Security Council (2015). Resolution 2242. Available online at: https://digitallibrary.un.org/record/807245?ln=enandv=pdf (accessed April 15, 2024).

[B7] van Douwen N. van den Brink M. Benschop Y. (2022). Badass marines: resistance practices against the introduction of women in the Dutch military. Gender Work Org. 29, 1443–1462. 10.1111/gwao.12835

[B8] Williams J. Yates S. Connor J. (2024). Creating a new pathway for change in the military using gender as process. Gender Work Org. 31, 211–226. 10.1111/gwao.13049

